# Valorizing *Salicornia brachiata* Seeds: A Rich Source of Polyphenols, Essential Fatty Acids, and Therapeutic Bioactivities

**DOI:** 10.1155/ijfo/2940708

**Published:** 2026-04-02

**Authors:** Poornima Jeewanthi, Nilaksha Navod, Sanath Madhushantha, Renuka Attanayake, Dinum Perera, Priyani Paranagama

**Affiliations:** ^1^ Department of Chemistry, Faculty of Science, University of Kelaniya, Kelaniya, Sri Lanka, kln.ac.lk; ^2^ Department of Plant and Molecular Biology, Faculty of Science, University of Kelaniya, Kelaniya, Sri Lanka, kln.ac.lk; ^3^ Department of Bioprocess Technology, Faculty of Technology, Rajarata University of Sri Lanka, Mihintale, Sri Lanka, rjt.ac.lk

**Keywords:** antibacterial, antioxidants, enzyme inhibition, phytochemicals, *Salicornia brachiata* seed

## Abstract

*Salicornia brachiata* is an extreme halophyte that grows in saline marshes and produces seeds rich in phytochemicals and associated bioactivities. This study is aimed at comprehensively evaluating the phytochemical composition, fatty acid profile, and multifunctional bioactivities of *S. brachiata* seeds. Dried powdered *S. brachiata* seeds were subjected to sequential extraction using hexane (HE), dichloromethane (DE), and methanol (ME) in order. The fatty acid profile of HE was analyzed using GC‐MS. Total phenolic content (TPC), total flavonoid content (TFC), condensed tannin content (CTC), and antioxidant activity of each extract were determined. Enzyme inhibitory potential was evaluated against *α*‐amylase, *α*‐glucosidase, lipase, angiotensin‐converting enzyme, and urease. Anti‐inflammatory activity and antibacterial activity were assessed for *S. brachiata* seed powder extracts. The results revealed that ME has the highest quantities of TPC, TFC, and CTC. GC‐MS analysis of HE revealed linoleic acid (53.32%) as the dominant fatty acid, with an unsaturated‐to‐saturated ratio of 2.36:1, indicating strong nutritional and bioactive potential. ME showed the lowest IC_50_ for DPPH and ABTS radical scavenging assays: 32.13 ± 0.97 and 75.11 ± 2.17 * μ*g/mL, respectively. HE expressed a potent inhibition for antidiabetic activity compared to acarbose. The lowest IC_50_ for lipase inhibition among extracts was shown by ME (241.67 ± 24.91 * μ*g/mL). DE showed strong antihypertensive activity (78.78*%* ± 1.08*%*) at 0.5 mg/mL, though less effective than captopril (91.85*%* ± 1.23*%*). HE and DE expressed lower IC_50_ values: 32.40 ± 2.21 and 12.43 ± 1.31 * μ*g/mL, respectively, in the urease inhibitory assay, highlighting their higher potency compared to thiourea with an IC_50_ value of 72.23 ± 3.76 * μ*g/mL. DE and ME showed less potency than aspirin in the anti‐inflammatory assay. Antibacterial efficacy of *S. brachiata* seed powder extracts demonstrated a broad spectrum of defense against tested bacteria. Therefore, *S. brachiata* seeds exhibit multifunctional bioactivities, making them an ideal supplement for fortified foods as well as an effective nutraceutical.

## 1. Introduction

Halophytic plants, particularly those from the genus *Salicornia* (family: Amaranthaceae), are increasingly recognized for their ecological resilience [1, 2] and potential as bioactive‐rich resources for pharmaceutical [3] and nutraceutical applications [4]. These succulent, salt‐tolerant species not only thrive in hypersaline soils where conventional crops fail [5] but also offer a diverse array of phytochemicals, including phenolic acids [6, 7], flavonoids [6, 8], tannins [8], and unsaturated fatty acids (UFAs) [7, 9, 10]. This biochemical richness is largely attributed to salinity‐induced stress, which activates defense mechanisms in *Salicornia* and results in the synthesis of polyphenols and other antioxidant metabolites, ultimately enhancing the plant′s bioactive profile [11]. Within this genus, species such as *S. europaea*, *S. bigelovii*, *S. herbacea*, and *S. ramosissima* have been extensively studied, especially their aerial parts for antioxidants [12–14], antimicrobial [8], anti‐inflammatory [12, 15], and even anticancer properties [16]. However, seeds, despite being rich in oil and phenolics, remain significantly underexplored.

The biological activities, such as antioxidant activity, antimicrobial activity, anti‐inflammatory activity, and anticancer activity, are largely attributed to the rich phytochemical composition of the aerial tissues, including polyphenols, flavonoids, and other secondary metabolites. In contrast, the seeds, despite being rich in bioactive compounds, have historically received less scientific attention. Available studies indicate that *Salicornia* seeds contain moderate to high levels of phenolic and flavonoid compounds [10, 19, 20] and are also rich in proteins [17]. Extracts from these seeds have demonstrated notable radical scavenging [19, 22], antimicrobial [21], and cytotoxic activities [17]. For instance, ethyl acetate fractions of *S. herbacea* seeds have shown strong antioxidant and antibacterial effects, along with selective cytotoxicity against colon cancer cell lines [17]. Similarly, gas chromatography‐mass spectrometry (GC‐MS) analyses of *S. bigelovii* seed oil have revealed a high proportion of UFAs, approximately twice the content of saturated fatty acids (SFAs), highlighting their potential nutritional and bioactive value [10]. More recently, an investigation into *S. brachiata* seed oil has demonstrated promising antidiabetic and antiobesity potential through a combination of *in silico*, *in vitro*, and *in vivo* approaches [22].

*S. brachiata*, endemic to tropical coastal regions of South Asia, has demonstrated promising bioactivities in its aerial parts. A recent study conducted by our group showed that methanol extracts of freeze‐dried aerial parts exhibited the highest phenolic content (43.68 ± 0.04 mg GAE/g DW) and strong antioxidant activity (2,2‐diphenyl‐1‐picrylhydrazyl [DPPH] IC_50_ = 8.72 ± 0.50 * μ*g/mL and 2,2 ^′^‐azino‐bis [3‐ethylbenzothiazoline‐6‐sulfonic acid] [ABTS] IC_50_ = 19.49 ± 0.76 * μ*g/mL), along with notable anti‐inflammatory and antibacterial effects [23]. These findings highlighted the bioactive potential of *S. brachiata*′s aboveground biomass. However, despite the biochemical richness and oil‐bearing nature of its seeds, their phytochemical composition and multifunctional bioactivities remain largely uncharacterized. It is noteworthy that a large portion of the current research on *Salicornia* seeds has concentrated on seed oils from species other than *S. brachiata*, specifically *S. bigelovii*, *S. herbacea*, and *S. europaea*. For instance, *S. bigelovii* has been described as an oilseed halophyte with a fatty acid profile dominated by linoleic acid (~73%–75%) and 26%–33% seed oil [10, 24, 25]. Fatty acid profiling and fundamental nutritional parameters are the primary focus of these studies, with limited attention paid to specific bioactive compounds or multitargeted bioactivities. Conversely, there is still a lack of research on *S. brachiata* seeds. Aside from a recent study that used *in vitro*, *in vivo*, and *in silico* methods to describe its potential to prevent diabetes and obesity [26], no study has yet undertaken a comprehensive investigation of its polyphenol subclasses, full phytochemical composition, or multifunctional bioactivities. The novelty and importance of the current study are amply demonstrated by this gap. Specifically, no previous study has thoroughly assessed the fatty acid composition, polyphenol subclasses derived from seeds, or their inhibitory effects on microbial growth, inflammatory activity, and metabolic enzymes. The wider functional valorization of *S. brachiata* as a halophytic resource is constrained by this knowledge gap.

The present study is aimed at providing the first comprehensive evaluation of the phytochemical composition and multifunctional bioactivities of *S. brachiata* seeds. Sequential solvent extraction was employed to obtain fractions of varying polarity, which were analyzed for total phenolic content (TPC), total flavonoid content (TFC), condensed tannin content (CTC), antioxidant activity, and fatty acid composition via GC‐MS. In addition, the extracts were assessed for their ability to inhibit key metabolic and inflammatory enzymes, *α*‐amylase, *α*‐glucosidase, lipase, urease, and angiotensin‐converting enzyme (ACE), alongside anti‐inflammatory and antibacterial properties. This multitargeted approach was designed to determine the seeds′ potential as a sustainable source of bioactive compounds with nutraceutical relevance.

## 2. Materials and Methods

### 2.1. Chemicals and Reagents

Hexane, dichloromethane, methanol, and ethanol solvents were purchased from Sigma‐Aldrich (St. Louis, Missouri, United States). Anhydrous sodium sulfate, sodium hydroxide, sodium dihydrogen orthophosphate dihydrate, sodium nitrite, and disodium hydrogen orthophosphate were purchased from Loba Chemie (Mumbai, India). Trichloroacetic acid, DPPH, ABTS, ferric chloride, sodium carbonate, aluminum chloride hexahydrate, and Folin–Ciocalteu (FC) reagent were purchased from Sigma‐Aldrich (St. Louis, Missouri, United States), while the sodium bicarbonate and sulfuric acid were purchased from VWR International (Poole, England). Vanillin was obtained from the Research Lab Fine Chem (Mumbai, India). The standards, butylated hydroxytoluene (BHT), gallic acid, catechin, acarbose, orlistat, thiourea, amoxicillin, and captopril, were purchased from Sigma‐Aldrich (St. Louis, Missouri, United States). The enzymes *α*‐amylase, *α*‐glucosidase, lipase, ACE, and urease, as well as the substrates p‐nitrophenyl‐*α*‐D‐glucopyranoside (PNPG), starch, p‐nitrophenyl acetate, urea, and hippuryl–histidyl–leucine (HHL), were also purchased from Sigma‐Aldrich (St. Louis, Missouri, United States). Nutrient agar and nutrient broth were purchased from HiMedia (Maharashtra, India).

### 2.2. Collection of Seeds

Mature *S. brachiata* plants at the senescence stage, bearing dispersal‐ready seeds, were manually uprooted from Karamba, Puttalam, Sri Lanka (7°58 ^′^28.0 ^″^ N, 79°48 ^′^42.5 ^″^ E) in late October 2024. The collected plants were transported in labeled polythene bags to the Department of Chemistry, University of Kelaniya, Sri Lanka, within the same day. Samples were air‐dried at room temperature (~30°C) for 5 days. Subsequently, seeds were manually separated from the seed‐bearing stem segments, and the surrounding perianth tissues were removed. Cleaned seeds were stored at −20°C for further analysis. All necessary approvals for plant collection were obtained from the relevant authorities.

### 2.3. Extract Preparation

The freeze‐dried *S. brachiata* seeds were ground and sieved through a 1‐mm mesh (ASTM E11‐20) to ensure uniform particle size for optimal extraction. The weight of the powdered *S. brachiata* seeds taken for sequential extraction was 50.23 g. Sequential solvent extraction was carried out to fractionate metabolites based on polarity, under continuous agitation (200 rpm) at 25^°^ *C* ± 2^°^ *C* using an orbital shaker (M TOS 6048F, TopsCien Instrument, Ningbo, China). Hexane (1:10 *w*/*v*, 24 h) was first used to extract nonpolar compounds (hexane extract [HE]), followed by dichloromethane (1:10 *w*/*v*, 24 h) for medium‐polar constituents (dichloromethane extract [DE]), and finally methanol (1:10 *w*/*v*, 24 h) for polar metabolites (ME). The extracts were filtered through Whatman Grade 1 paper, dried with anhydrous Na_2_SO_4_, and concentrated under reduced pressure at 40°C using a rotary evaporator (Rotavapor R‐300, Büchi Labortechnik AG, Flawil, Switzerland). Crude extracts were stored at −20°C until further analysis.

### 2.4. Determination of TPC, TFC, and CTC

TPC of *S. brachiata* seed powder extracts was quantified using the FC assay based on a previously published procedure with minor modifications [27]. Reaction mixture contained 20 *μ*L of gallic acid standard or *S. brachiata* seed powder extract with 100 *μ*L of 1:10 diluted FC reagent. The mixture was incubated for 7 min at ambient temperature, followed by the addition of 80 *μ*L of 7.5% sodium carbonate. The solution was protected from light and incubated for 2 h to facilitate the redox‐driven chromogenic reaction. Absorbance was measured at 765 nm using a microplate photometer (Multiscan FC, Thermo Fisher Scientific, Massachusetts, United States). Results were expressed as milligrams of gallic acid equivalents per gram of dry weight (mg GAE g^−1^ DW), reflecting the extract′s phenolic richness.

TFC quantification was done based on a previously published procedure with minor modifications [28, 29]. An aliquot of 100 *μ*L of *S. brachiata* seed powder extract or catechin standard was mixed with 30 *μ*L of 5% sodium nitrite, and after 5 min, 50 *μ*L of 2% aluminum chloride was added, followed by 50 *μ*L of 1 M NaOH after 6 min. The mixture was incubated for 10 min at 25°C, and the absorbance was measured at 510 nm. Results were expressed as milligrams of catechin equivalents per gram dry weight (mg CE g^−1^ DW), indicating flavonoid abundance.

CTC was assessed based on a previously established method with slight modifications [30, 31]. An aliquot of 20 *μ*L of *S. brachiata* seed powder extract was mixed with 150 *μ*L of 4% vanillin (in methanol) and 150 *μ*L concentrated sulfuric acid. Absorbance was measured at 500 nm after 15 min of incubation. Results were expressed as milligrams of catechin equivalents per gram dry weight (mg CE g^−1^ DW).

### 2.5. GC‐MS Analysis of Fatty Acid Content

The fatty acid composition of HE of *S. brachiata* seed powder was determined by GC‐MS after derivatization to fatty acid methyl esters (FAMEs). The procedure for saponification and methylation was adapted from a previously established method with minor modifications [32, 33]. Saponification was proceeded by mixing HE of *S. brachiata* seed powder with 50 mL of 3% alcoholic KOH under reflux at 80°C for 6 h. After cooling, the mixture was extracted thrice with hexane, and the combined extracts were washed with distilled water, dried over anhydrous Na_2_SO_4_, and concentrated under reduced pressure at 40°C. For derivatization, free fatty acids were refluxed in methanol with H_2_SO_4_ at 80°C for 3 h to form FAMEs, which were then extracted with hexane, dried, and concentrated. FAME analysis was performed via GC‐MS (Agilent 7890B GC/5977A MSD, Agilent Technologies, Santa Clara, California, United States) equipped with an HP‐88 capillary column (100 *m* × 0.25 *m* *m* × 0.20 *μ* *m*). The oven program was 50°C (2 min), 50°C–240°C (4°C/min), and 240°C (15 min) with helium as carrier gas (1 mL/min). Compound identification was performed by mass spectral matching against the National Institute of Standards and Technology (NIST) 17 Mass Spectral Database.

### 2.6. Functional Group Characterization by ATR‐FTIR

ATR‐FTIR spectroscopy was carried out to characterize the functional groups present in HE of *S. brachiata* seeds. Spectra were recorded using a PerkinElmer Spectrum 2 FTIR spectrometer equipped with an attenuated total reflectance (ATR) accessory. Each sample was directly placed onto the diamond ATR crystal, and spectra were collected in the range of 500–4000 cm^−1^. Each spectrum represented the average of 16 scans acquired at a resolution of 4 cm^−1^. Background spectra were recorded prior to each measurement and automatically subtracted. All analyses were performed at room temperature.

### 2.7. Bioactivity Profiles

#### 2.7.1. *In Vitro* Antioxidant Activity

Extracts of *S. brachiata* seed powder were assessed for antioxidant activity by two radical‐based assays (DPPH and ABTS) and one metal‐related assay: ferric reducing antioxidant power (FRAP) assay, by taking the standard positive control as BHT. DPPH radical scavenging activity was assessed based on a previously established method with slight modifications [34, 35]. A freshly prepared 0.25 mM DPPH solution in methanol (40 *μ*L) was mixed with 160 *μ*L of *S. brachiata* seed powder extracts or BHT at varying concentrations. The reaction mixture was incubated in the dark at room temperature for 15 min, and the absorbance was measured at 517 nm. The percentage of DPPH^•^ scavenging activity was calculated using Equation (1):

(1) DPPH•scavenging effect %=A0−A1 A0×100.

*A*_0_ and *A*_1_ correspond to the absorbances at 517 nm of the radical DPPH^•^ in the absence and presence of an antioxidant, respectively.

ABTS radical scavenging activity was assessed based on a previously established method [36]. To perform the ABTS radical scavenging assay, ABTS was first dissolved in distilled water to obtain a 7 mM stock solution. This solution was then mixed with 2.45 mM potassium persulfate and incubated in the dark at room temperature for 12–16 h to generate the ABTS radical cation (ABTS^•+^). The resulting solution was diluted with ethanol to achieve an absorbance of 0.700 ± 0.020 at 734 nm. Prior to analysis, the diluted ABTS^•+^ solution was equilibrated at 30°C. In the assay, 1.0 mL of ABTS^•+^ solution was mixed with 10 *μ*L of *S. brachiata* seed powder extracts or BHT at varying concentrations. The mixture was incubated for 6 min, and the absorbance was recorded at 734 nm. The percentage of ABTS^•+^ scavenging activity was calculated using Equation (2):

(2) ABTS•+ scavenging effect %=A0−A1 A0×100.

*A*_0_ and *A*_1_ correspond to the absorbances at 734 nm of the radical ABTS^•+^ in the absence and presence of an antioxidant, respectively.

The FRAP assay was assessed based on a previously established method with slight modifications [37, 38]. The reaction mixture for the FRAP assay was prepared by mixing 10 *μ*L of *S. brachiata* seed powder extracts or BHT at varying concentrations with 25 *μ*L of 0.2 M sodium phosphate buffer (pH 6.6) and 25 *μ*L of 1% potassium ferricyanide. After incubation at 45°C for 20 min, the reaction was terminated by adding 25 *μ*L of 10% trichloroacetic acid. The mixture was then diluted with 85 *μ*L of deionized water, followed by the addition of 17 *μ*L of 0.1% ferric chloride. After 10 min, absorbance was measured at 700 nm, and the interpretation of the results was done using absorbance values.

#### 2.7.2. *α*‐Amylase Inhibition Activity

*α*‐Amylase inhibition assay was conducted by following a previously published method with minor adjustments [39, 40]. To screen for *α*‐amylase inhibition potential, varying concentrations of *S. brachiata* seed powder extracts or acarbose (as the positive control) were preincubated with 100 *μ*L of *α*‐amylase enzyme (1 U/mL) for 10 min. Then, 100 *μ*L of 1% (*w*/*v*) starch solution was added, and the mixture was incubated at 37°C for 10 min. The reaction was terminated by adding 100 *μ*L of DNS reagent (containing sodium potassium tartrate, sodium hydroxide, and 3,5‐dinitrosalicylic acid), followed by heating the mixture in a boiling water bath for 10 min. Absorbance was measured at 540 nm, and *α*‐amylase inhibition was calculated using Equation (3):

(3) α‐Amylase inhibition %=A0−A1 A0×100.

*A*_0_ and *A*_1_ correspond to the absorbances at 405 nm in the absence and presence of *S. brachiata* seed powder extract or acarbose, respectively.

#### 2.7.3. *α*‐Glucosidase Inhibition Activity

The *α*‐glucosidase inhibition activity was assessed by following a previously published method with minor adjustments [41, 42]. The yeast *α*‐glucosidase (1 U/mL) was used as the enzyme and 5 mM PNPG as the substrate, and both were prepared in 50 mM potassium phosphate buffer (pH 6.8). An aliquot of 50 *μ*L of the enzyme solution was mixed with 50 *μ*L of varying concentrations of *S. brachiata* seed powder extracts or acarbose (as the positive control) and preincubated at 25°C for 5 min. Following preincubation, 200 *μ*L of the substrate solution was added, and the reaction mixture was incubated for another 10 min. The absorbance was measured at 405 nm, and inhibition was calculated using Equation (4):

(4) α‐Glucosidase inhibition %=A0−A1 A0×100.

*A*_0_ and *A*_1_ correspond to the absorbances at 540 nm in the absence and presence of *S. brachiata* seed powder extract or acarbose, respectively.

#### 2.7.4. Lipase Inhibition Activity

Lipase inhibition activity was assessed by following a previously published method with minor adjustments [43]. An aliquot of 50 *μ*L of lipase enzyme (1 mg/mL in Tris buffer, pH 7.4) was preincubated with varying concentrations of *S. brachiata* seed powder extracts or orlistat (as the positive control) for 10 min. Then, an aliquot of 50 *μ*L of p‐nitrophenyl acetate (4 mM in Tris buffer, pH 8.0) was added to each well, and the mixture was incubated at 37°C for 30 min. The absorbance was measured at 405 nm, and the percentage inhibition of lipase activity was calculated using Equation (5):

(5) Lipase inhibition %=A0−A1A0×100.

*A*_0_ and *A*_1_ correspond to the absorbances at 405 nm in the absence and presence of *S. brachiata* seed powder extract or orlistat, respectively.

#### 2.7.5. Anti‐Inflammatory Activity

Anti‐inflammatory activity was assessed by following a previously published method with minor adjustments [44, 45]. The human red blood cell (HRBC) membrane stabilization assay was conducted to evaluate anti‐inflammatory activity. Blood was collected from a healthy volunteer, centrifuged at 3000 rpm, and washed three times with normal saline. The washed blood cells were reconstituted to a 10% *v*/*v* suspension in normal saline. The reaction mixture contained 1 mL of varying concentrations of *S. brachiata* seed powder extracts or aspirin (as the positive control) and 0.1 mL of the 10% red blood cell suspension. The reaction mixtures were incubated in a water bath at 56°C for 30 min, cooled, and centrifuged at 3000 rpm for 10 min. The absorbance of the supernatants was measured at 540 nm, and the percentage inhibition of hemolysis was calculated using Equation (6):

(6) Inhibition of hemolysis %=A0−A1A0×100.

*A*_0_ and *A*_1_ correspond to the absorbances at 560 nm in the absence and presence of *S. brachiata* seed powder extract or aspirin, respectively.

#### 2.7.6. ACE Inhibition Activity

The ACE inhibition assay was conducted following a previously established method with slight modifications [46–48]. Initially, an aliquot of 40 *μ*L of *S. brachiata* seed powder extracts or captopril (as the positive control) was mixed with 100 *μ*L of phosphate buffer (100 mM, pH 8.3) containing 0.2 M NaCl and 7.0 mM HHL as the substrate. The reaction was initiated by adding 60 *μ*L of ACE solution (0.1 U/mL), followed by incubation at 37°C for 30 min. To terminate the reaction, 30 *μ*L of 1 M HCl was added. The hippuric acid formed was extracted using 850 *μ*L of ethyl acetate, and the mixture was centrifuged at 1200 × *g* for 15 min. The organic layer was collected, and ethyl acetate was removed through heat evaporation. The extracted residue was then dissolved in 2.5 mL of distilled water, and the absorbance was measured at 228 nm. The percentage inhibition of ACE activity was calculated using Equation (7):

(7) ACE inhibition %=A0−A1A0×100.

*A*_0_ and *A*_1_ correspond to the absorbances at 228 nm in the absence and presence of *S. brachiata* seed powder extract or captopril, respectively.

#### 2.7.7. Urease Inhibition Activity

The urease inhibition assay was conducted following a previously established method with slight modifications [49]. In the urease inhibitory activity assay, the reaction mixture consisted of urea (125 mM, 80 *μ*L) as the substrate, 20 *μ*L of *S. brachiata* seed powder extract or thiourea (as the positive control), and phosphate buffer (pH 7.4), and the enzymatic reaction was initiated by adding urease enzyme (80 *μ*L). After 30 min of incubation, ammonia concentration was determined by adding 500 *μ*L of Solution A (0.50 g phenol and 2.50 mg sodium nitroprusside in 100 mL distilled water) and 500 *μ*L of Solution B (0.50 g sodium hydroxide and 840 *μ*L of 5% sodium hypochlorite in 100 mL distilled water). The mixture was incubated at 37°C for 30 min, and absorbance was measured at 625 nm. The percentage inhibition of urease activity was calculated using Equation (8):

(8) Urease inhibition %=A0−A1A0×100.

*A*_0_ and *A*_1_ correspond to the absorbances at 625 nm in the absence and presence of *S. brachiata* seed powder extract or thiourea, respectively.

#### 2.7.8. Antibacterial Activity Assay

The antibacterial activity assay was conducted following a previously established method with slight modifications [50, 51]. Antibacterial activity was evaluated using the agar well diffusion method against *Staphylococcus aureus* (ATCC 25923), *Bacillus subtilis* (MTCC 121), *Pseudomonas aeruginosa* (ATCC 15442), and *Escherichia coli* (ATCC 25922). *S. brachiata* seed powder extracts were tested at three concentrations (0.5, 1.0, and 2.0 mg/mL). Amoxicillin (0.1 mg/mL) served as the standard positive control. Bacterial cultures were spread uniformly on agar plates, and 6‐mm wells were punched into the solidified agar. Each well received 100 *μ*L of *S. brachiata* seed powder extract/positive control at the appropriate concentration. Plates were incubated at 37°C for 24 h. Antibacterial activity was quantified by measuring the inhibition zone diameter (mm) in three different directions and calculating the average by using Equation (9):

(9) Antibacterial activity mm=D1+D2+D33.

*D*_1_, *D*_2_, and *D*_3_ refer to the diameters of the inhibition zone taken in three different directions.

#### 2.7.9. Statistical Analysis

All experiments were performed in triplicate, and the results were subjected to one‐way analysis of variance (ANOVA) using SPSS software Version 16.0 (IBM Corp., Armonk, New York, United States) to evaluate treatment effects. When significant differences were observed (*p* < 0.05), Tukey′s honestly significant difference (HSD) test was applied for pairwise comparisons among treatment means. *I* *C*_50_ values were calculated by nonlinear regression curve fitting using GraphPad Prism Version 8 (GraphPad Software, San Diego, California, United States). Results are expressed as *m* *e* *a* *n* ± *s* *t* *a* *n* *d* *a* *r* *d* deviation (SD) in tables, while SD values are represented as error bars in figures.

## 3. Results and Discussion

### 3.1. Determination of TPC, TFC, and CTC

**Figure 1:**
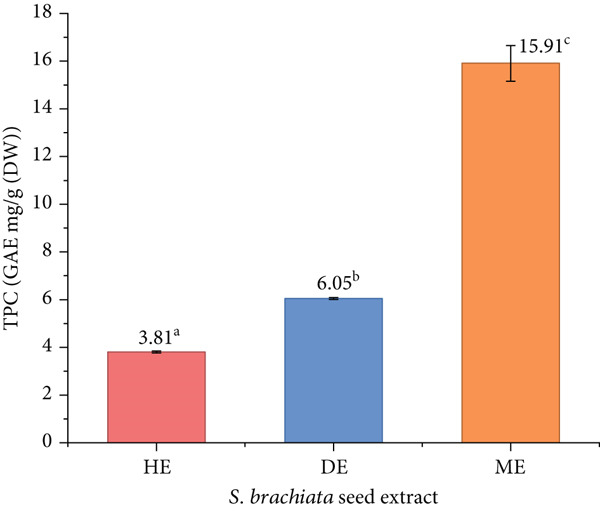
Variation of total phenolic content (TPC) of hexane extract (HE), dichloromethane extract (DE), and methanol extract (ME) of *S. brachiata* seed powder. Each bar represents mean ± SD (*n* = 3). Letters a, b, and c were used to compare statistical significance (*p* < 0.05).

**Figure 2:**
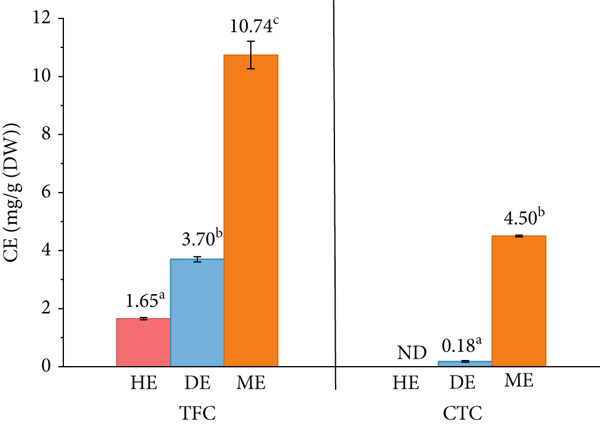
Variation of total flavonoid content (TFC) and condensed tannin content (CTC) of hexane extract (HE), dichloromethane extract (DE), and methanol extract (ME) of *S. brachiata* seed powder. Each bar represents mean ± SD (*n* = 3). Letters a, b, and c were used to compare statistical significance (*p* < 0.05).

The phytochemical composition of *S. brachiata* seed powder extract, specifically TPC, TFC, and CTC, depicted in Figures 1 and 2, respectively, was markedly influenced by the polarity of the extraction solvent used. Among the three solvent extracts, the ME exhibited the highest concentrations of all three compounds, with a TPC of 15.91 ± 0.75 mg GAE/g DW, a TFC of 10.74 ± 0.47 mg CE/g DW, and a CTC of 4.50 ± 0.03 mg CE/g DW. In contrast, the DE and HE yielded progressively lower values, with the HE showing no detectable levels of condensed tannins.

These findings underscore the predominantly polar nature of phenolics, flavonoids, and tannins in *S. brachiata* seeds, reflecting their greater solubility in polar solvents such as methanol. Similar solvent‐dependent extraction patterns have been reported in other *Salicornia* species. For instance, in *S. herbacea*, methanol and ethyl acetate fractions exhibited significantly higher TPC and TFC values compared to nonpolar hexane extracts [17]. Likewise, ethanol extracts of *S. bigelovii* seeds recorded lower TPC and TFC values (4.5 and 2.9 mg/g, respectively) than the methanolic extract of *S. brachiata* in this study [10]. Furthermore, extractions with petroleum ether in *S. bigelovii* and *S. europaea* yielded minimal phenolic and flavonoid contents [18], reinforcing the limited efficiency of nonpolar solvents in isolating polar phytoconstituents.

The presence of condensed tannins exclusively in the methanol and dichloromethane fractions reinforces the pivotal role of solvent polarity in the selective recovery of phytochemicals due to their hydrophilic nature [6] and underscores the biochemical complexity of *S. brachiata* seeds. Condensed tannins, also known as proanthocyanidins, are high molecular weight polyphenolic compounds that typically exhibit limited solubility in nonpolar solvents due to their polar functional groups and polymeric structure [52]. Their absence in the HE and clear enrichment in polar and semipolar solvent fractions reflect the affinity of these compounds for solvents capable of hydrogen bonding and dipole–dipole interactions [53, 54]. This selective solubility not only highlights the importance of choosing appropriate extraction solvents when targeting specific phytochemical classes but also suggests that *S. brachiata* seeds harbor structurally diverse metabolites requiring tailored extraction strategies. The ability to isolate such compounds effectively has significant implications for downstream applications in food science, pharmacology, and natural product chemistry.

This pronounced accumulation of phenolic compounds may be attributed to the species′ adaptation to saline and arid environments [55, 56]. Abiotic stressors such as salinity and osmotic stress are known to induce secondary metabolite production in halophytes, thereby enhancing the biosynthesis of antioxidant‐rich compounds, including flavonoids and tannins [55, 57].

The polyphenolic levels found in *S. brachiata* seeds fall within or above the values previously reported in the literature when compared to published phytochemical ranges for other *Salicornia* species. The strong solvent‐dependent extraction efficiency, particularly with methanol, suggests that *S. brachiata* seeds can serve as a potent source of natural antioxidants and bioactives. These findings not only validate the pharmacological potential of *S. brachiata* but also contribute to the broader understanding of *Salicornia* as a genus rich in biofunctional seed chemistry.

### 3.2. GC‐MS Analysis of Fatty Acid Content

The GC‐MS analysis of the HE from *S. brachiata* seed powder (Table 1) revealed a fatty acid composition dominated by polyunsaturated fatty acids (PUFAs), with linoleic acid (C18:2, 9,12‐octadecadienoic acid) accounting for 53.32% of the total fatty acid content. The SFAs detected included palmitic acid (C16:0, 14.52%) and stearic acid (C18:0, 4.67%) as major components, along with minor amounts of lauric acid (C12:0, 0.13%), myristic acid (C14:0, 0.24%), caproic acid (C6:0, 0.16%), margaric acid (C17:0, 0.12%), arachidic acid (C20:0, 0.88%), behenic acid (C22:0, 1.17%), and tricosanoic acid (C23:0, 0.69%), resulting in a total SFA content of 22.58%. The high proportion of linoleic acid, a polyunsaturated omega‐6 fatty acid, is particularly noteworthy due to its well‐established hypocholesterolemic effects. Comparative studies indicate that linoleic acid is more effective than oleic acid in reducing total cholesterol levels, including lowering low‐density lipoprotein (LDL), very low‐density lipoprotein (VLDL), and high‐density lipoprotein (HDL) cholesterol concentrations, although interindividual variability may influence the degree of response [58]. Moreover, recent meta‐analyses suggest that linoleic acid decreases LDL cholesterol slightly more efficiently than oleic acid, supporting its role in cardiovascular health and lipid regulation [60]. The exclusive presence of linoleic acid contributed to the total UFA content of 53.32%, entirely comprised of PUFAs in this profile. In terms of nutritional evaluation, the PUFA/SFA ratio was calculated to be 2.36. According to dietary lipid recommendations, this *P* *U* *F* *A*/*S* *F* *A* *r* *a* *t* *i* *o* > 2 suggests a potentially beneficial lipid profile for reducing plasma and liver lipid accumulation, specifically reducing plasma VLDL concentrations [59].

**Table 1:** GC‐MS analysis of the fatty acid content in the hexane extract (HE) of *S. brachiata* seeds.

**Compound name**	**Common name**	**Retention time (min)**	**Fatty acid content (%)**
Dodenoic acid	Lauric acid	19.98	0.13
Tetradecanoic acid	Myristic acid	22.61	0.24
Hexanoic acid	Caproic acid	24.91	0.16
Hexadecanoic acid	Palmitic acid	25.25	14.52
Hetadecinoic acid	Margaric acid	26.17	0.12
Octadecanoic acid	Stearic acid	19.30	4.67
Eicosanoic acid	Arachidic acid	29.52	0.88
Docosanoic acid	Behenic acid	31.95	1.17
Tricosanoic acid	Tricosylic acid	34.35	0.69
9,12‐Octadecadienoic acid	Linoleic acid	27.37	53.32
Total saturated fatty acid content (∑SFA) (%)			22.58
Total unsaturated fatty acid content (∑UFA) (%)			53.32
∑SFA:∑UFA ratio			1:2.36

The predominance of linoleic acid in *S. brachiata* seeds is consistent with trends observed in other *Salicornia* species. For instance, *S. bigelovii* seeds exhibited linoleic acid contents ranging from 74.66% to 79.49%, oleic acid from 12.33% to 16.83%, and SFAs such as palmitic and stearic acids between 7% and 8.5% and 1.24% and 1.69%, respectively [60]. In another study, different extraction methods applied to *S. bigelovii* revealed linoleic acid levels ranging from 34.9% to 36.5%, with total UFA content between 63.3% and 64.7% and the highest yields obtained via ultrasonic extraction using ethanol [10]. These methodological variations underscore the influence of solvent polarity and technique on fatty acid yield and composition.

Comparable lipid compositions have also been reported for *S. herbacea* seeds, where linoleic acid accounted for 43.73%, followed by oleic acid (19.81%), arachidic acid (13.52%), palmitic acid (11.84%), lauric acid (0.04%), myristic acid (0.13%), margaric acid (0.14%), stearic acid (3.07%), behenic acid (2.52%), and tricosanoic acid (0.03%) [20]. Additionally, this seed oil was rich in tocopherols and sterols and exhibited notable antioxidant capacity, suggesting multifunctional health‐promoting potential. While the linoleic acid content in *S. herbacea* is lower than that of *S. brachiata* in the present study, the presence of structurally similar fatty acids supports the nutritional significance of *Salicornia* seeds more broadly.

Similar fatty acid trends have also been documented in *S. fruticosa*, where seed oil showed 17.4% linoleic acid, 56.58% oleic acid, and a total UFA content of 78.05% [102]. In *S. europaea*, environmental salinity influenced fatty acid composition, with seeds from low‐salinity soils exhibiting higher linoleic acid content (up to 65.1%) and lower oleic acid content, while high‐salinity soils showed the reverse pattern [103]. Importantly, a recent study analyzing the HE of *S. brachiata* seed oil reported a linoleic acid content of 38.85%, with palmitic acid (14.94%) and stearic acid (4.10%) as the predominant SFAs [26]. Although this is lower than the 53.32% found in the present work, such variation may stem from differences in extraction methods, ecotypic traits, and environmental conditions. These findings underscore the influence of both intrinsic and extrinsic factors on fatty acid composition in halophytes.

The high PUFA content in *S. brachiata* aligns with the health benefits attributed to halophytic oils, including cardioprotective, anti‐inflammatory, and hypolipidemic effects [102, 103]. Linoleic acid, an essential fatty acid, contributes to maintaining cell membrane integrity, modulating inflammatory responses, and supporting metabolic health [61, 62]. Moreover, a high UFA/SFA ratio (approximately 2.36:1 in this study) is considered advantageous for dietary fat quality [63, 64].

Taken together, the lipidomic profile of *S. brachiata* seeds highlights their potential value as a source of essential fatty acids for dietary supplementation and functional food development. The dominance of linoleic acid, low SFA content, and absence of trans fats reinforce the nutritional and bioactive promise of *S. brachiata* seed oil.

### 3.3. Functional Group Characterization by ATR‐FTIR

**Figure 3:**
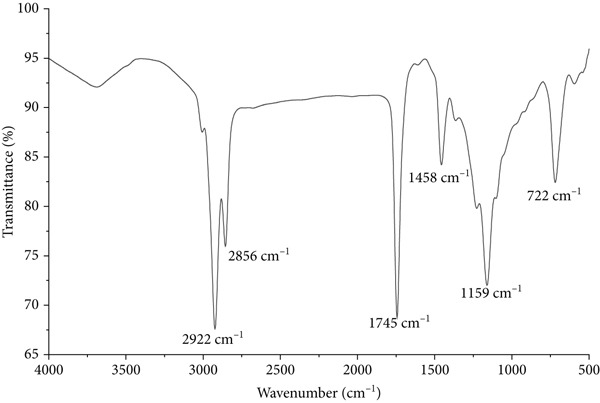
ATR–FTIR spectrum of the hexane extract (HE) of *S. brachiata* seeds.

FTIR analysis of HE of *S. brachiata* seeds (Figure 3) revealed absorption bands characteristic of long‐chain fatty acids and lipid esters, consistent with the GC‐MS fatty acid profile. A strong band at 1745 cm^−1^ corresponds to the C=O stretching vibration of ester carbonyl groups, indicative of triglycerides and fatty acid esters [65]. Prominent peaks at 2922 and 2856 cm^−1^ represent asymmetric and symmetric C–H stretching of methylene groups, confirming the presence of long aliphatic chains [65]. The band at 1458 cm^−1^ is attributed to CH_2_ bending vibrations, while the peak at 1159 cm^−1^ corresponds to C–O stretching of ester linkages [65]. A characteristic band near 722 cm^−1^ reflects (CH_2_)_n_ rocking, typically observed in SFAs and UFAs with long hydrocarbon chains [65]. Collectively, these spectral features strongly support the lipid‐rich, nonpolar composition of HE.

### 3.4. Bioactivity Profiles

#### 3.4.1. *In Vitro* Antioxidant Activity

**Figure 4:**
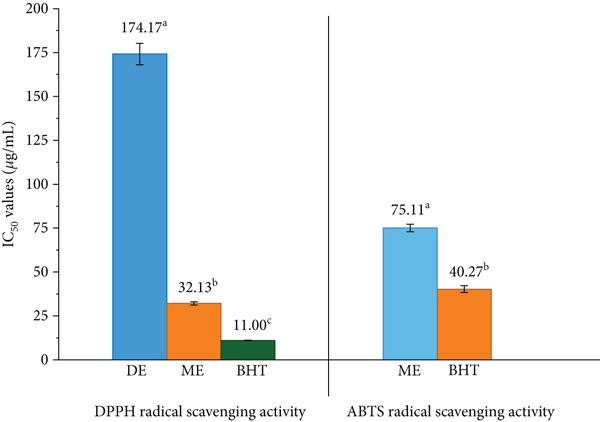
DPPH radical scavenging activity (IC_50_ values) of dichloromethane extract (DE) and methanol extract (ME) of *S. brachiata* seed powder and standard positive control, butylated hydroxytoluene (BHT) and ABTS radical scavenging activity (IC_50_ values) of ME of *S. brachiata* seed powder and standard positive control, BHT. Each bar represents mean ± SD (*n* = 3). Letters a, b, and c were used to compare statistical significance (*p* < 0.05).

**Figure 5:**
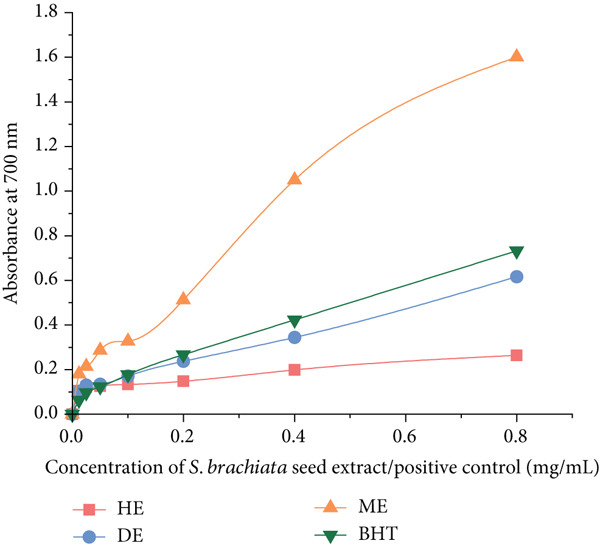
Ferric reducing antioxidant power of hexane extract (HE), dichloromethane extract (DE), and methanol extract (ME) of *S. brachiata* seed powder and standard positive control, butylated hydroxytoluene (BHT). Each bar represents mean ± SD (*n* = 3).

The antioxidant potential of *S. brachiata* seed powder extracts, as evaluated by DPPH and ABTS radical scavenging assays and the FRAP assay, is presented in Figures 4 and 5, respectively. Among the tested fractions, the ME exhibited the strongest radical scavenging activity, with *I* *C*_50_ values of 32.13 ± 0.97 * μ*g/mL (DPPH) and 75.11 ± 2.17 * μ*g/mL (ABTS). DE demonstrated moderate DPPH activity (174.17 ± 6.08 * μ*g/mL), while HE was largely inactive in both assays, with *I* *C*_50_ values exceeding 1000 *μ*g/mL. For comparison, the standard antioxidant BHT exhibited lower *I* *C*_50_ values, confirming its high potency.

These antioxidant results are consistent with the phytochemical profiles described earlier in this study, where ME exhibited the highest concentrations of total phenolics, flavonoids, and condensed tannins. The pronounced radical scavenging activity of ME can be attributed to the high polarity of these bioactive compounds, which are more efficiently extracted into polar solvents. In addition to its free radical scavenging potential, ME also demonstrated the highest FRAP, indicating superior electron‐donating capacity. The combined high performance across all three antioxidant assays reinforces the extract′s richness in polyphenolic antioxidants. In contrast, HE, which showed the lowest phytochemical content, also demonstrated the weakest antioxidant activity.

These findings align with prior studies on *S. herbacea*, where polar solvent extracts such as methanol and ethyl acetate consistently outperformed less polar fractions like hexane, ether, and water in radical scavenging assays [17]. Specifically, ethyl acetate fractions demonstrated the lowest IC_50_ values among all solvent systems for both DPPH (0.18 mg/mL) and ABTS (0.87 mg/mL) assays, while methanol fractions showed moderate activity (IC_50_ ~1–1.7 mg/mL). Ether and aqueous extracts also displayed weaker antioxidant capacity, confirming the selectivity of polyphenol solubility and function.

Furthermore, a previous study assessed the antioxidant properties of *S. herbacea* seed oil using the ABTS assay [20], reporting that increasing the concentration of methanol‐extracted oil from 100 to 300 *μ*g/mL enhanced ABTS radical scavenging activity from 50.2% to 71.8%. Similarly, increasing the concentration of hexane‐extracted oil from 100 to 300 *μ*g/mL resulted in an increase in activity from 35.3% to 60.1%. In the present study, a comparable trend was observed: ABTS radical scavenging activity of *S. brachiata* seed powder extracts increased from 8.8% to 93.7% for MEs and from 15.5% to 35.5% for HEs across concentrations ranging from 16 to 1000 *μ*g/mL. These results further substantiate the polarity‐dependent gradient of antioxidant activity observed in the current work.

#### 3.4.2. Antidiabetic Activity

**Figure 6:**
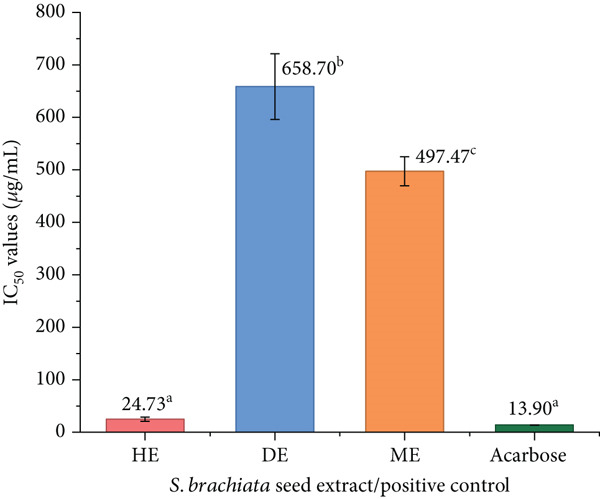
*α*‐Amylase inhibitory activity (IC_50_ values) of hexane extract (HE), dichloromethane extract (DE), and methanol extract (ME) of *S. brachiata* seed powder and standard positive control, acarbose. Each bar represents mean ± SD (*n* = 3). Letters a, b, and c were used to compare statistical significance (*p* < 0.05).

**Figure 7:**
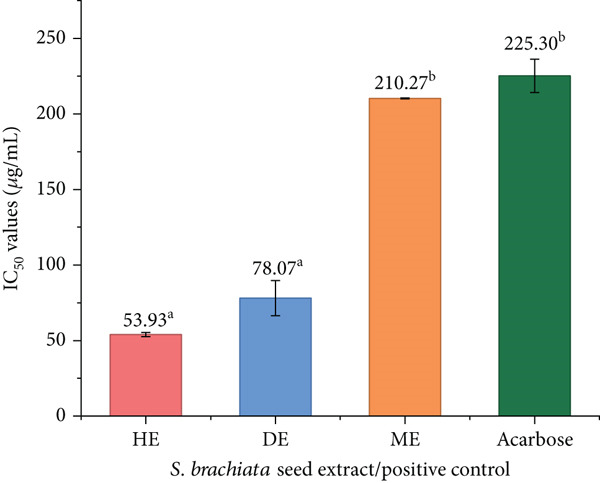
*α*‐Glucosidase inhibitory activity (IC_50_ values) of hexane extract (HE), dichloromethane extract (DE), and methanol extract (ME) of *S. brachiata* seed powder and standard positive control, acarbose. Each bar represents mean ± SD (*n* = 3). Letters a, b, and c were used to compare statistical significance (*p* < 0.05).

The inhibitory activities of the HE, DE, and ME of *S. brachiata* seed powder against *α*‐amylase and *α*‐glucosidase are presented in Figures 6 and 7, respectively. Among the tested extracts, HE exhibited the strongest enzyme inhibition, with *I* *C*_50_ values of 24.73 ± 4.11 * μ*g/mL for *α*‐amylase and 53.93 ± 1.39 * μ*g/mL for *α*‐glucosidase. Notably, HE outperformed the standard antidiabetic drug acarbose in *α*‐glucosidase inhibition (*I* *C*_50_: 225.30 ± 11.04 * μ*g/mL), underscoring its potent bioactivity. In contrast, DE and ME showed significantly weaker inhibition, suggesting that the major active compounds responsible for enzyme inhibition are likely nonpolar in nature and efficiently extracted by hexane.

Total phenolic, flavonoid, and tannin levels were not correlated with *α*‐amylase and *α*‐glucosidase inhibition, in contrast to antioxidant activity. Despite having a low polyphenolic content, HE′s comparatively higher activity indicates the presence of nonpolar bioactive components. The GC‐MS results of this study, which demonstrate that HE has high concentrations of UFAs, especially linoleic acid, a known modulator of enzymes that break down carbohydrates in earlier research, corroborate this [66]. Therefore, enzyme inhibition in HE appears to be driven predominantly by lipophilic metabolites rather than phenolics.

These findings are consistent with a recent study on *S. brachiata* seed oil extracted using hexane, which reported dose‐dependent *α*‐glucosidase inhibition, with inhibition rates of 16.80%, 19.00%, and 30.00% at concentrations of 16, 32, and 48 mg/mL, respectively. Compared to these values, HE in the present study demonstrated significantly higher potency, achieving IC_50_ at just 53.93 *μ*g/mL, reinforcing the enhanced activity of our optimized HE [26].

Similar solvent polarity‐dependent trends have been reported in other halophytic seeds as well. For example, *Chenopodium quinoa* seed powder extracts prepared with varying ethanol concentrations exhibited improved inhibition of both *α*‐glucosidase and *α*‐amylase enzymes. Reported IC_50_ values ranged from 48.67 ± 0.6 to 92.38 ± 0.66 mg/mL for *α*‐glucosidase and 61.36 ± 0.58 to 105.73 ± 1.60 mg/mL for *α*‐amylase, substantially higher than those obtained in the current study [67].

The biochemical behavior of long‐chain UFAs, which interact with digestive enzymes through hydrophobic insertion into the enzyme surface and partially distort the catalytic pocket, is responsible for HE′s potent *α*‐amylase and *α*‐glucosidase inhibitory effects [68]. Previous enzyme kinetic studies indicate that linoleic acid and other UFAs can inhibit carbohydrate‐digesting enzymes, often through competitive [66] or mixed‐type mechanisms [69]. These effects are generally attributed to hydrophobic interactions between the fatty acid chain and enzyme surface regions, while the polar carboxyl group may contribute to weaker interactions near the catalytic site. These interactions reduce substrate accessibility and disrupt conformational flexibility of *α*‐amylase and *α*‐glucosidase.

#### 3.4.3. Antilipase Activity

**Figure 8:**
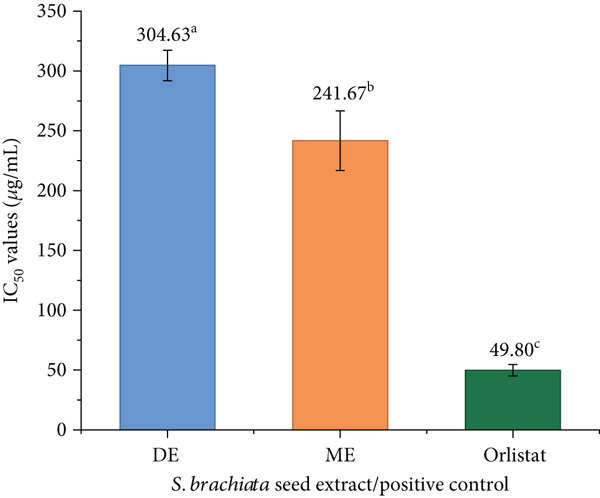
Lipase inhibitory activity (IC_50_ values) of dichloromethane extract (DE) and methanol extract (ME) of *S. brachiata* seed powder and standard positive control, orlistat. Each bar represents mean ± SD (*n* = 3). Letters a, b, and c were used to compare statistical significance (*p* < 0.05).

The results of the lipase inhibition assay, including *I* *C*_50_ values, are depicted in Figure 8. In contrast to the inhibition patterns observed for *α*‐amylase and *α*‐glucosidase, lipase inhibition followed a distinct trend. The ME exhibited the strongest inhibitory effect among the test samples, with an *I* *C*_50_ value of 241.67 ± 24.91 * μ*g/mL. Although this is markedly less potent than the standard lipase inhibitor orlistat (*I* *C*_50_: 49.80 ± 4.76 * μ*g/mL), it still indicates moderate bioactivity.

DE demonstrated intermediate activity (IC_50_: 304.63 ± 12.74 * μ*g/mL), whereas HE was largely inactive, with negligible inhibition. These findings clearly indicate that lipase inhibition in *S. brachiata* seeds is primarily linked to polar phenolic constituents, in contrast to *α*‐glucosidase inhibition, where nonpolar fatty acids contribute significantly. Pancreatic lipase is known to be inhibited by polyphenols like flavonoids and tannins through hydrophobic interactions and hydrogen bonding with the catalytic pocket of the enzyme [70]. Therefore, the strong activity observed in ME is consistent with its high polyphenolic load, while the weak activity of HE confirms that lipophilic fatty acids in this extract do not significantly contribute to lipase inhibition.

Given the increasing interest in natural lipase inhibitors as potential agents for obesity management, these findings highlight the potential of *S. brachiata* seeds as a functional ingredient for nutraceutical development. However, further isolation and characterization of the active constituents are warranted to confirm their mechanisms of action and efficacy.

This study′s lipase inhibition pattern is consistent with known phenolic–enzyme interaction mechanisms. Multiple hydrogen bonds and *π*–*π* hydrophobic stacking between the enzyme′s lipophilic residues and aromatic phenolic rings mediate this interaction, resulting in conformational constraints that lower catalytic efficiency [70].

#### 3.4.4. Antihypertension Activity

**Figure 9:**
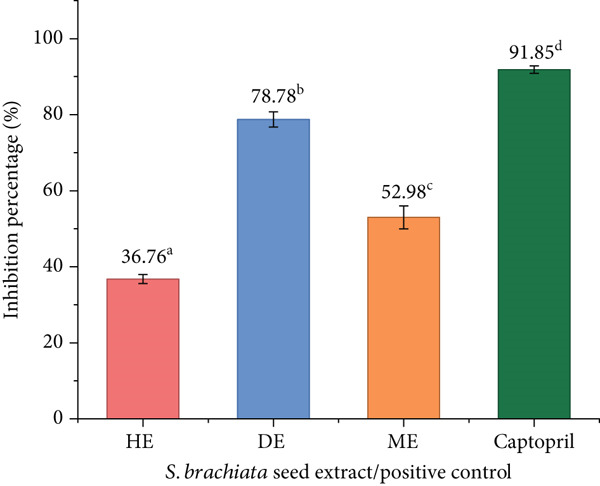
Angiotensin‐converting enzyme inhibitory activity of hexane extract (HE), dichloromethane extract (DE), and methanol extract (ME) of *S. brachiata* seed powder and standard positive control, captopril, at the concentration of 0.5 mg/mL. Each bar represents mean ± SD (*n* = 3). Letters a, b, and c were used to compare statistical significance (*p* < 0.05).

The ACE inhibitory activity of *S. brachiata* seed powder extracts was assessed at a concentration of 0.5 mg/mL, with results shown in Figure 9. Among the three tested fractions, the DE exhibited the highest inhibition (78.78*%* ± 1.08*%*), followed by the ME (52.98*%* ± 2.45*%*) and HE (36.76*%* ± 1.45*%*). Although none of the extracts matched the potency of the standard drug captopril (91.85*%* ± 1.23*%*), the DE fraction demonstrated a substantial inhibitory effect, suggesting the presence of semipolar bioactive compounds with significant ACE inhibitory potential.

ACE inhibition is one of the most effective bioactive strategies for managing hypertension, as it reduces the formation of Angiotensin II, a potent vasoconstrictor. In vitro assay employed in this study utilizes HHL as a substrate, where ACE hydrolyzes HHL to form hippuric acid, which is then quantified to determine the degree of enzyme inhibition [71].

These findings align with previous reports on other halophytes. For instance, a recent study on *Mesembryanthemum crystallinum* demonstrated that polyphenol‐rich extracts exhibited potent ACE inhibitory activity, achieving up to 90.5% inhibition at 1 mg/mL. The high activity was primarily attributed to flavonoids such as apigenin and luteolin derivatives, highlighting the role of specific phenolic compounds in modulating ACE function [72]. Comparatively, it is found that the *n*‐butanol extract of *Suaeda physophora* was identified as the active fraction, demonstrating significant ACE inhibitory activity with an 83.5% inhibition rate at 100 mg/mL and a 70.1% inhibition rate at 50 mg/mL, attributing this effect to the presence of flavonoid and phenolic compounds [73]. Similarly, *Artemisia scoparia* has been shown to have ACE inhibitory activity, with studies indicating lower ACE activity and Angiotensin II content in the serum of spontaneously hypertensive rats treated with its extracts, suggesting the involvement of phenolics [74]. Studies on *Salicornia ramosissima* have shown its antihypertensive activity, with oven‐dried and freeze‐dried samples demonstrating similar effects at concentrations of 24.56 and 18.96 mg/mL, respectively, attributed in part to its phenolic compounds [75]. In an in vivo study using 2K1C hypertensive rats, a single daily oral dose of 10 mg/kg of lyophilized aqueous extract of *Tribulus terrestris* fruit for 4 weeks significantly decreased systolic blood pressure. This antihypertensive effect was associated with a notable reduction in ACE activity in various tissues, including the aorta, heart, kidney, lung, and serum, with the most prominent reduction observed in the kidney [76]. Another previous study investigated the antihypertensive effects of *Tribulus terrestris* in spontaneously hypertensive rats, demonstrating that both ME and aqueous extract dose‐dependently reduced blood pressure. Notably, the aqueous fraction exhibited greater potency than the methanolic fraction at all tested doses. Their findings suggested that these antihypertensive effects stemmed from a direct arterial smooth muscle relaxation, potentially mediated by nitric oxide release [77]. Similarly, *Sarcocornia perennis*, a halophytic species within the same family as *Salicornia*, showed promising in vivo antihypertensive effects in a human pilot trial, where consumption of *Sarcocornia*‐based salt substitutes led to significant reductions in both systolic blood pressure and arterial stiffness [78].

Similarly, ACE inhibition showed no direct correlation with the phenolic, flavonoid, or tannin content across extracts. The highest ACE inhibition in DE indicates involvement of semipolar metabolites, particularly various flavonoids [79], fatty acid esters [80], and phytosterol derivatives [81], which have been previously reported as natural ACE modulators. These results indicate that ACE inhibition in *S. brachiata* seeds is chemically driven by nonphenolic constituents. Continued phytochemical investigation and compound‐level characterization will be necessary to identify the specific ACE inhibitors present in *S. brachiata* seeds and evaluate their bioactive potential through *in vivo* models.

The mechanistic interactions between semipolar phytochemicals and the ACE catalytic system can account for the strong ACE inhibition seen in the DE extract. Peptide hydrolysis depends on the central Zn^2+^ ion in the ACE active site, which is coordinated by the HEXXH zinc‐binding motif [82]. It is known that a number of plant metabolite classes, especially different flavonoids and midpolar phenolic esters, inhibit ACE, possibly by chelating the catalytic Zn^2+^ ion and by occupying hydrophobic subpockets close to the active site [83].

#### 3.4.5. Antiurease Activity

**Figure 10:**
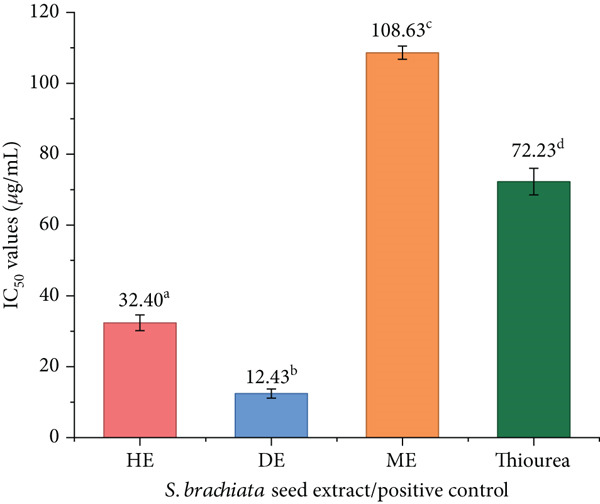
Urease inhibitory activity (IC_50_ values) of hexane extract (HE), dichloromethane extract (DE), and methanol extract (ME) of *S. brachiata* seed powder and standard positive control, thiourea. Each bar represents mean ± SD (*n* = 3). Letters a, b, and c were used to compare statistical significance (*p* < 0.05).

The urease inhibitory potential of *Salicornia brachiata* seed powder extracts is depicted in Figure 10, revealing notable differences among the solvent fractions. Among the three extracts, the DE demonstrated the most potent activity, with an *I* *C*_50_ value of 12.43 ± 1.31 * μ*g/mL, surpassing both the ME (108.63 ± 1.87 * μ*g/mL) and the standard inhibitor thiourea (72.23 ± 3.76 * μ*g/mL). These results suggest that midpolar compounds extracted by dichloromethane play a central role in urease inhibition.

Interestingly, this trend is consistent with findings from other halophytes. A recent study on *Atriplex nitens* demonstrated that its methanolic seed powder extract exhibited exceptional urease inhibition, with an IC_50_ of 6.58 ± 0.48 * μ*g/mL, outperforming thiourea (16.68 ± 1.12 * μ*g/mL) by a significant margin [84]. Although the IC_50_ value of *S. brachiata* DE in the present study is slightly higher than that of *A. nitens*, it remains more effective than its own positive control, highlighting strong inhibitory potential.

The high potency of DE of *S. brachiata* seed powder confirms the presence of bioactive constituents with targeted enzyme‐inhibitory activity and further underscores the critical role of solvent polarity in isolating efficacious urease‐inhibiting compounds. This strong urease inhibitory activity observed for DE can be mainly due to the presence of semipolar metabolites such as flavonoids [85], coumarins [86], alkyl phenols, and midpolar terpenoids, which are known urease inhibitors in earlier phytochemical studies. Thus, urease inhibition in the DE extract is likely mediated by semipolar secondary metabolites. This pronounced inhibitory effect is especially relevant considering urease′s role in the pathogenesis of gastrointestinal disorders such as peptic ulcers and urinary tract infections, where the enzyme catalyzes the hydrolysis of urea into ammonia, leading to mucosal damage and facilitating microbial colonization. The ability of plant‐derived compounds to inhibit urease thus presents a compelling bioactive strategy, particularly in light of the toxicity concerns associated with conventional inhibitors like thiourea.

The known molecular interactions between semipolar phytochemicals and the active site architecture of urease provide a mechanistic explanation for the strong urease inhibition seen in the DE fraction. There is a binuclear Ni^2+^ center in urease [87], coordinated by histidine and aspartic acid molecules, which are essential for catalyzing urea hydrolysis [88]. Several classes of plant metabolites identified in DE, such as several flavonoids [89], coumarins [86], alkyl phenols, and midpolar terpenoids, are reported to inhibit urease by chelating the Ni^2+^ ions, forming hydrogen bonds with active‐site residues, or blocking the mobile flap that controls substrate entry [90].

#### 3.4.6. Anti‐inflammatory Activity

**Figure 11:**
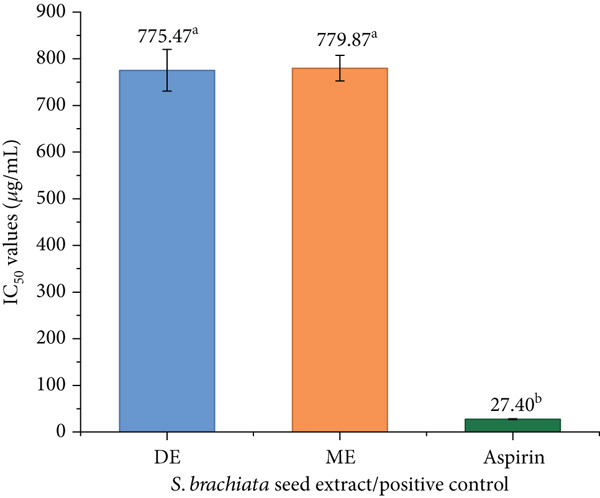
Anti‐inflammatory activity of dichloromethane extract (DE) and methanol extract (ME) of *S. brachiata* seed powder and standard positive control, aspirin. Each bar represents mean ± SD (*n* = 3). Letters a, b, and c were used to compare statistical significance (*p* < 0.05).

The anti‐inflammatory potential of extracts of *S. brachiata* seed powder was evaluated using the HRBC membrane stabilization assay, with results depicted in Figure 11. ME and DE showed anti‐inflammatory activity with *I* *C*_50_ values of 779.87 ± 27.56 and 775.47 ± 44.38 * μ*g/mL, respectively. In contrast, aspirin, a standard anti‐inflammatory control, displayed an *I* *C*_50_ of 27.40 ± 1.12 * μ*g/mL, indicating substantially higher potency compared to *S. brachiata* seed powder extracts. This lower efficacy of ME can likely be attributed to its content of polyphenols and flavonoids, known to inhibit proinflammatory pathways such as cyclooxygenase (COX) and lipoxygenase (LOX) [91]. Similarly, DE′s comparable activity suggests the presence of semipolar compounds with significant anti‐inflammatory potential.

These findings are consistent with composition activity relationships observed in other *Salicornia* species [91]. In *S. europaea*, the O‐methylated flavone acacetin has been shown to significantly suppress proinflammatory mediators, including inducible nitric oxide synthase (iNOS) and COX‐2, reducing oxidative stress and inflammation *in vitro* [92]. Likewise, the isoflavonoid Irilin B, isolated from *S. europaea*, demonstrated both anti‐inflammatory and antioxidative effects in BV‐2 microglial cells, suggesting potential for neuroprotection [93, 94]. *S. ramosissima* and *S. herbacea* aerial extracts contain additional bioactive phenolics such as ferulic acid, coumarins, and flavonoid glycosides like quercetin‐3‐glucoside and isorhamnetin‐3‐glucoside, which contribute to reactive oxygen species (ROS) scavenging and inflammatory modulations [91, 95].

These data emphasize that the anti‐inflammatory activity of *Salicornia* species is strongly linked to their polyphenolic profiles. In *S. brachiata*, the moderate activity observed in polar extracts supports the presence of similar phenolic constituents, albeit perhaps at lower concentrations or with different structures than those found in *S. europaea*. To more precisely determine the anti‐inflammatory contributors, future work should employ bioassay‐guided fractionation to isolate and characterize compounds, such as flavonoids, phenolic acids, and saponins, and directly evaluate their effects on COX, LOX, cytokine production, and other inflammatory mediators.

The capacity of some phytochemicals to directly interact with the lipids and proteins of the erythrocyte membrane can be mechanistically linked to the membrane stabilization seen in the HRBC assay. Through hydrogen bonding with polar head groups and hydrophobic interactions with acyl chains, a variety of polyphenols and flavonoids found in ME and DE are known to intercalate into phospholipid bilayers, improving membrane rigidity, preventing osmotic lysis, and suppressing heat‐induced structural disruption [96].

#### 3.4.7. Antibacterial Activity Assay

The antibacterial efficacy of *S. brachiata* seed powder extracts was assessed against *Escherichia coli*, *Staphylococcus aureus*, *Bacillus subtilis*, and *Pseudomonas aeruginosa* using the well diffusion method, with the corresponding inhibition zone diameters summarized in Table 2. ME demonstrated the most pronounced activity, particularly against *Staphylococcus aureus* (20.10 ± 0.30 mm inhibition zone at 2000 *μ*g/mL) and *Pseudomonas aeruginosa* (30.30 ± 0.20 mm inhibition zone at 2000 *μ*g/mL). In the case of *P. aeruginosa*, the inhibition zone produced by ME exceeded that of the standard antibiotic amoxicillin at 100 *μ*g/mL (17.40 ± 0.65 mm), suggesting promising antibacterial potency at higher concentrations. DE also exhibited notable antibacterial activity, especially against *Bacillus subtilis*, while HE showed minimal efficacy, with activity limited to high concentrations against *P. aeruginosa*. Its membrane permeability and efflux properties may limit the uptake of the extract constituents, as indicated by the lack of activity against *E. coli* [97]. The inhibition of *P. aeruginosa*, however, suggests that Gram‐negative bacteria′s response varies depending on the species and is not exclusively influenced by the outer membrane [98].

**Table 2:** Antibacterial activity of hexane extract (HE), dichloromethane extract (DE), and methanol extract (ME) of *S. brachiata* seed powder and standard positive control, amoxicillin, by the well diffusion method.

**Bacteria strain**	**Extract**	**Mean zone of inhibition (mm)**			
		***S. brachiata* seed extracts (*μ*g/mL)**			**Standard (amoxicillin) 100 *μ*g/mL**
		**500**	**1000**	**2000**	
*Escherichia coli*	HE	NS	NS	NS	30.00 ± 1.98
	DE	NS	NS	NS	
	ME	NS	NS	16.60 ± 0.68	

*Staphylococcus aureus*	HE	9.30 ± 0.20^a^	9.70 ± 0.20^a^	14.30 ± 0.20^a^	31.04 ± 1.77
	DE	NS	12.30 ± 0.20^b^	18.30 ± 0.20^b^	
	ME	10.00 ± 0.10^a^	14.70 ± 0.30^c^	20.10 ± 0.30^c^	

*Bacillus subtilis*	HE	NS	NS	NS	14.11 ± 1.19
	DE	13.20 ± 0.70^a^	18.40 ± 0.33^a^	14.20 ± 0.70^a^	
	ME	10.60 ± 0.20^b^	11.90 ± 0.30^a^	13.40 ± 0.80^a^	

*Pseudomonas aeruginosa*	HE	12.00 ± 0.20^a^	14.40 ± 0.60^a^	18.70 ± 0.70^a^	17.40 ± 0.65
	DE	11.60 ± 0.80^a^	11.80 ± 0.70^a^	12.30 ± 1.10^b^	
	ME	NS	22.30 ± 0.50^b^	30.30 ± 0.20^c^	

*Note:* Values represent the mean ± SD in triplicate (*n* = 3). Letters a, b, and c were used to compare statistical significance (*p* ≤ 0.05) in the same column of respective concentration of *S. brachiata* seed powder extracts tested for the given specific bacterial strain (*n* = 3).

Abbreviation: NS, no sensitivity.

The observed variation in activity among extracts is likely due to differences in the polarity of the solvents used, which influence the extraction of bioactive compounds [99]. These results indicate that the antibacterial potential of *S. brachiata* seeds is likely associated with the presence of polar and midpolar secondary metabolites, including phenolics and flavonoids, as previously highlighted. The broad‐spectrum antibacterial activity of ME is consistent with its rich phenolic and flavonoid profile [100], as similar mechanisms have been described for other phenolic‐rich halophyte extracts that disrupt bacterial membranes, interfere with enzymatic function, or induce oxidative stress [101]. The efficacy of DE against *Bacillus subtilis* also points toward the contribution of moderately polar constituents such as terpenoids or alkaloid‐like compounds.

These findings are consistent with previous work on *S. herbacea*, where ethanol seed powder extracts produced inhibition zones of 9.5 mm against *S. aureus* and 3.5 mm against *E. coli* [21]. The superior antibacterial activity of *S. brachiata* ME and DE suggests a broader spectrum or higher concentration of antimicrobial phytoconstituents, underscoring their bioactive potential. Furthermore, the selective and differential activity of each extract highlights the value of solvent‐specific extraction strategies to harness distinct antibacterial mechanisms.

Overall, these results provide compelling evidence for the multifunctional bioactivity of *S. brachiata* seed powder extracts. ME demonstrated strong antioxidant and antibacterial properties, while HE and DE exhibited notable enzyme inhibitory activities relevant to antidiabetic, antiulcer, and antihypertensive applications. The polarity‐dependent distribution of these activities reflects the diverse phytochemical makeup of the seeds. Collectively, the findings support the potential of *S. brachiata* seeds as a valuable source of bioactive compounds for use in functional foods and plant‐based bioactives targeting oxidative stress, metabolic dysfunction, and microbial infections.

## 4. Conclusions

This study presents the first comprehensive investigation of the phytochemical composition, fatty acid profile, and multifunctional bioactivities of *S. brachiata* seeds. The results demonstrate that solvent polarity significantly influences the recovery of bioactive constituents, with methanol proving most effective in extracting phenolics, flavonoids, and condensed tannins. These polar extracts, particularly the methanolic fraction, exhibited strong antioxidant activity across multiple assays, validating the correlation between polyphenolic richness and radical scavenging capacity.

GC‐MS analysis of the hexane fraction revealed a lipid profile dominated by linoleic acid (53.32%) and an overall high UFA content, surpassing most of the previously reported values for other *Salicornia* species. This lipid composition underscores the nutritional and bioactive promise of *S. brachiata* seed oil, particularly its potential contribution to omega‐6 intake and cardiovascular health.

Bioactivity assays further revealed that each solvent extract possessed distinct functional attributes. HE showed superior inhibitory activity against *α*‐glucosidase, often exceeding the performance of the standard drug acarbose, while DE excelled in urease and ACE inhibition. In contrast, ME displayed strong lipase inhibitory potential and broad‐spectrum antibacterial efficacy, notably surpassing the standard antibiotic in selected assays. Also, both DE and ME displayed nearly the same anti‐inflammatory activity potential.

Collectively, these findings confirm the multifunctional health‐promoting potential of *S. brachiata* seeds. Their rich phytochemical and lipid profiles, coupled with diverse enzyme inhibitory and antimicrobial activities, support their valorization as a novel, sustainable source of nutraceutical ingredients. These results provide a scientific foundation for further exploration of *S. brachiata* in the development of functional foods, dietary supplements, and plant‐based bioactives aimed at managing oxidative stress, metabolic disorders, and microbial infections.

The current research proves *Salicornia* seeds to be an excellent bioresource with antioxidant, antibacterial, and enzyme inhibitory activities. Phytochemical analysis showed high bioactive compound content, which was responsible for the observed bioactivities. The bioactivities exhibited strong antioxidant potential, signified by expanding their usage as oxidative stress alleviators. Antibacterial screening indicated that the extracts possessed a broad‐spectrum activity, which indicates great promise in antimicrobial formulations. Moreover, the enzyme inhibitory studies emphasized remarkable inhibition against some prominent enzymatic activities, which might directly or indirectly cause different disorders. *S. brachiata* seeds display multifunctional bioactivities, making them an alternative to fortified foods and nutraceuticals, and therefore warrant deeper study for *in vivo* efficacy and safety to support their medical potential in actual clinical scenarios.

## Disclosure

All authors have critically reviewed and approved the final version of the manuscript for submission.

## Conflicts of Interest

The authors declare no conflicts of interest.

## Author Contributions

Priyani Paranagama, Dinum Perera, and Renuka Attanayake were responsible for conceptualization, comprehensive research design, overall project supervision, and critical review and editing of the manuscript. In addition to acquiring the research funding, Dinum Perera critically reviewed and drafted the introduction and discussion sections of the manuscript. Poornima Jeewanthi executed the experimental research, performed statistical data analysis, generated graphical representations, interpreted research findings, and was responsible for the primary drafting of the manuscript and iterative editing of manuscript versions, including the final submission. Nilaksha Navod and Sanath Madhushantha performed experimental investigations, primarily drafted the methodology section of the manuscript, and contributed to the interpretation of experimental results.

## Funding

This research was conducted as part of an industrial partnership between Rajarata University of Sri Lanka and Mega Plantations (Pvt.) Ltd., Sri Lanka. It was supported by the Science and Technology Human Resource Development Project, Ministry of Education, Sri Lanka, and funded by the Asian Development Bank (Grant No. CRG/R2/RJ1).

## Data Availability

The data that support the findings of this study are available from the corresponding authors upon reasonable request.
